# Use of a Resorbable Magnesium Membrane for Bone Regeneration After Large Radicular Cyst Removal: A Clinical Case Report

**DOI:** 10.3390/healthcare13091068

**Published:** 2025-05-06

**Authors:** Gabi Chaushu, Vadim Reiser, Eli Rosenfeld, Daya Masri, Liat Chaushu, Marija Čandrlić, Patrick Rider, Željka Perić Kačarević

**Affiliations:** 1 Department of Oral and Maxillofacial Surgery, Rabin Medical Center, Beilinson Campus, Petah Tikva 49100, Israel; gavrielce@clalit.org.il (G.C.); vadikraiser@gmail.com (V.R.); eliros@gmail.com (E.R.); dr.dayamasri@gmail.com (D.M.); 2 Department of Oral and Maxillofacial Surgery, The Maurice and Gabriela Goldschleger School of Dental Medicine, Faculty of Medicine, Tel Aviv University, Tel Aviv 6997801, Israel; 3 Department of Periodontology and Implant Dentistry, Faculty of Medicine, The Maurice and Gabriela Goldschleger School of Dental Medicine, Tel Aviv University, Tel Aviv 6997801, Israel; liat.natanel@gmail.com; 4 Department of Integrative Dental Medicine, Faculty of Dental Medicine and Health Osijek, Josip Juraj Strossmayer University of Osijek, 31 000 Osijek, Croatia; marija.candrlic@fdmz.hr; 5 Botiss Biomaterials GmbH, 15806 Zossen, Germany; patrick.rider@botiss.com; 6 Department of Anatomy, Histology, Embriology, Pathology Anatomy and Pathology Histology, Faculty of Dental Medicine and Health Osijek, Josip Juraj Strossmayer University of Osijek, 31 000 Osijek, Croatia

**Keywords:** cysts, odontogenic, case report, magnesium, bone regeneration, xenografts

## Abstract

Background: Periapical cysts are the most common odontogenic cysts, often resulting in large bone defects. Guided tissue regeneration techniques support tissue healing by means of membranes and bone grafts. The present case report evaluates for the first time clinical application of a resorbable magnesium membrane in guided bone regeneration (GBR) following cystectomy. Case report: A 35-year-old male patient presented with a large periapical cystic lesion in the maxillary anterior region. Treatment involved marsupialization followed by cyst enucleation and GBR using a resorbable magnesium membrane and bovine xenograft. The magnesium membrane served as a structural support to bridge the bony discontinuity in the palatal bone. Cone-beam computed tomography (CBCT) was used for diagnosis, treatment planning, and follow-up assessments. At 16 months post-treatment, CBCT imaging revealed significant bone regeneration, with restoration of the palatal contour and cortication of the palatal wall. Clinical examination showed asymptomatic teeth with normal mobility and optimal soft tissue healing. Conclusions: This case demonstrates the potential of resorbable magnesium membranes in managing large periapical defects, offering a promising alternative to traditional GBR materials by combining mechanical strength with complete resorption, therefore eliminating the need for membrane removal surgery. However, future studies on larger patient samples should focus on confirming the long-term outcomes of this approach and investigating patient-specific factors that are important in choosing effective treatment options.

## 1. Introduction

Periapical cysts, also known as radicular or periradicular cysts, are the most common odontogenic cysts, accounting for up to 60% of all cases, with a higher frequency of occurrence in the anterior maxilla and mandibular premolar regions [1,2,3]. Epidemiological studies report that radicular cysts occur in approximately 1.4% to 15% of all periapical radiolucencies, and in about 0.5% to 3% of all extracted teeth, although precise prevalence rates may vary across populations [4]. They originate from the inflammatory stimulation of epithelial cell rests of Malassez in the periodontal ligament. The formation of the cyst is usually triggered by pulpal necrosis or chronic periapical inflammation associated with a non-vital tooth [5]. Pathologically, these cysts are epithelial-lined cavities, with the most common histopathological finding being a lining of nonkeratinized stratified squamous epithelium [6]. Clinically, radicular cysts are typically asymptomatic and slow growing, often discovered incidentally during routine radiographic examinations. However, larger cysts may present with mild swelling, tenderness, or displacement of adjacent teeth. In some cases, secondary infection can cause pain, drainage, or even an acute inflammatory response [7]. If untreated, they can enlarge, causing progressive bone resorption and complicating treatment outcomes, particularly in young adults [5,8].

The treatment of cystic lesions of the jaw requires thorough preoperative evaluation to determine the appropriate approach based on the lesion’s size, location, and its relationship with surrounding anatomical structures [3]. For smaller lesions, non-surgical approaches such as endodontic therapy are often the first line of treatment, as they are conservative and carry minimal risk. However, when the cyst is large or there are significant changes in the periapical tissues that cannot be addressed through nonsurgical therapy, surgical interventions are indicated [8]. Among surgical treatments, cystectomy remains a gold standard method for eradicating cystic lesions. Cystectomy, also referred to as enucleation or the Partsch II operation, involves the complete removal of the cyst sac, followed by primary intention wound healing. The cyst is carefully separated from the bone along the inner bony surface to minimize damage to surrounding structures [7,9,10]. For larger cystic lesions, decompression or marsupialization is often a preliminary step. Decompression reduces cyst size by relieving internal pressure, while marsupialization further promotes bony apposition by creating a permanent opening for the cyst cavity to drain. Marsupialization is typically followed by enucleation after 3–6 months. While this approach minimizes damage to critical anatomical structures, prevents pathological fractures, and stimulates osteogenesis, it requires a prolonged treatment period and a second surgical intervention [11,12,13].

One of the major challenges following enucleation of large radicular cysts is managing the residual bone cavity and stability of the surrounding vital structures. To address these challenges, the use of biomaterials for regeneration such as bone grafts and membranes has guided bone regeneration (GBR) procedures in both medicine and dentistry to support hard and soft tissue healing. Also, GBR has been recommended as an adjunct to endodontic treatment and surgery to promote periapical tissue healing and improve treatment outcomes [14]. In that context, complete periapical healing requires the regeneration of alveolar bone, periodontal ligament cells, and cementum. However, the ingrowth of surrounding connective tissue into the osseous defect can compromise bone healing, especially if the one or more bone walls are missing [15]. By using membranes and other biomaterials, GBR techniques help prevent this collapse of connective tissues, hence promoting bone regeneration and stabilizing the defect [16].

Resorbable membranes, such as collagen membranes, are commonly used due to their biocompatibility and the advantage of eliminating the need for a second surgical procedure. However, their limited mechanical stability and rapid degradation can be challenging in cases of large defects [17]. Non-resorbable PTFE membranes provide superior structural integrity but require a second surgical removal after 4–6 weeks, increasing patient burden [18]. In response to these obstacles, magnesium-based membranes have recently emerged as a novel solution in GBR [19,20]. These membranes combine the advantages of resorbable and non-resorbable materials by providing good mechanical stability and being fully resorbable, eliminating the need for a second surgical intervention. Magnesium membranes have demonstrated promising results in various clinical scenarios by means of tissue regeneration. In addition, magnesium membranes have shown unique properties, such as promoting vascularization, supporting bone regeneration, and possessing antimicrobial effects [19,20,21,22].

Taking into account all the benefits of magnesium membranes, which in the first line combines full mechanical stability with complete resorbability, and the nature of bony defects such as cystic lesions, we proposed their use in large bony defects following cyst removal. In cases of extensive alveolar bone loss, maintaining structural integrity and stability is crucial for successful bone regeneration, and the form-stable nature of magnesium membranes offers a promising solution. To date, no published studies have reported the application of magnesium membranes specifically for GBR following cystectomy in large periapical cystic lesions. Therefore, this case report aims to present the use of magnesium membranes in GBR following cystectomy, demonstrating their potential in promoting bone regeneration in large post-cystectomy defects.

## 2. Case Report

### 2.1. Patient History and Clinical Findings

This case report adheres to the CARE (CAse REports) guidelines, with the corresponding checklist provided in the Supplementary Materials for reference [23]. The present case was selected for report due to its unique characteristics. After a discussion of diagnostic procedures and treatment options, the patient provided written informed consent for both the chosen treatment protocol and the publication of this case report. Due to the nature of the case report, formal Institutional Review Board approval was not required.

A 52-year-old male patient was referred to the dental clinic of the Oral and Maxillofacial Department at Rabin Medical Centre, Beilinson campus in Petach Tikva, Israel. The referral was prompted by an episode of severe swelling accompanied by purulent discharge from the anterior palate. The patient’s medical history was unremarkable, with no systemic diseases, no current medications, and no history of smoking. The presenting symptoms persisted for three weeks before resolution, which was achieved following a two-week course of antibiotic therapy. The prescribed regimen consisted of Amoxicillin 875 mg/Clavulanic acid 125 mg (Augmentin^®^, GlaxoSmithKline, Petach Tikva, Israel), administered twice daily.

- Extraoral Findings

The patient presented with facial symmetry and no evidence of facial swelling. Temporomandibular joint function was normal, with no limitations in mouth opening or associated pain. Submandibular lymph nodes were non-palpable and non-tender. No pathological findings were observed in the neck region. Facial sensory examination revealed no abnormalities.

- Intraoral Examination

Intraoral assessment revealed a localized palatal swelling in the region of the four maxillary incisors. No active purulent discharge was observed at the time of examination. The remaining oral mucosa appeared intact. All four maxillary incisors, namely 11, 12, 21 and 22 (FDI Notation System), exhibited no mobility; however, they failed to respond to electric pulp vitality testing. The patient demonstrated good oral hygiene and expressed a strong commitment to adhering to the proposed treatment plan.

- Radiographic Evaluation

**Figure 1:**
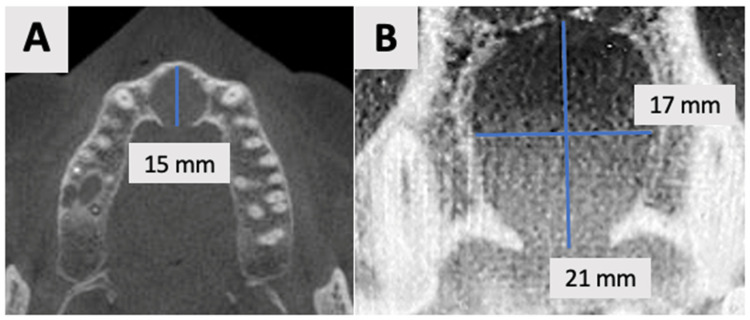
Radiographic images of the cyst at initial visit before marsupialization. (**A**) Axial, and (**B**) coronal views of the cyst center. Measurements indicate cyst dimensions: mesiodistal 17 mm × buccolingual 15 mm × height 21 mm.

Cone Beam Computed Tomography (CBCT) was performed using the NewTom GO 2D/3D*^®^* system (NewTom, Verona, Italy). Imaging was performed to assess the extent and characteristics of the lesion (Figure 1). The CBCT revealed a unilocular hypodense area palatal to the anterior four maxillary teeth, absence of palatal bony walls and intact buccal bony frame. Lesion dimensions were 17 mm (mesiodistal) × 15 mm (buccolingual) × 21 mm (height). These radiographic findings are consistent with the characteristics of a radicular cyst, which typically presents as a well-defined, unilocular radiolucency associated with the apex of a non-vital tooth. The absence of palatal bony walls suggests potential expansion or erosion of the palatal cortex.

Based on the clinical and radiographic findings, a preliminary clinical diagnosis of a radicular cyst associated with the maxillary incisors was established. The treatment plan was formulated to address both the pathological lesion and the resultant bony defect. The proposed treatment strategy involved a two-stage approach: initial marsupialization to reduce cyst size and promote bone formation, followed by cyst enucleation and GBR.

### 2.2. Marsupialization

**Figure 2:**
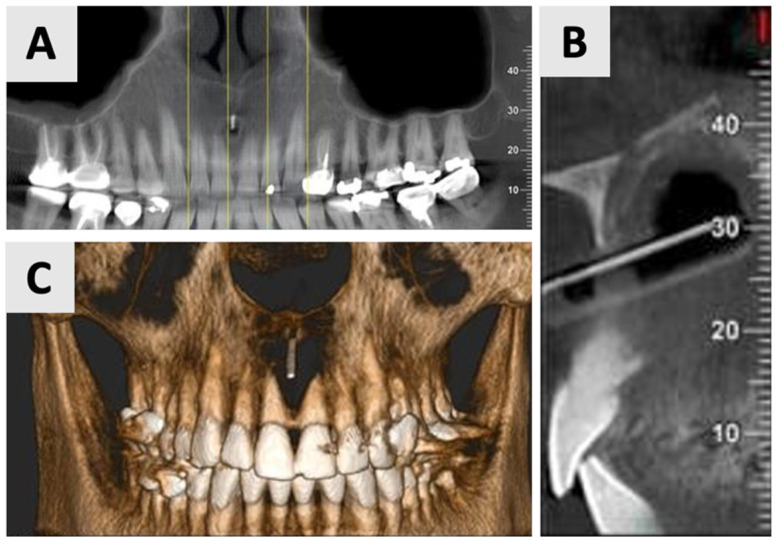
Radiographic evaluation of cyst six months following marsupialization. (**A**) Panoramic reconstruction illustrating the initial defect site prior to enucleation. (**B**) Cyst regression and improved bone density in the affected area. (**C**) Paraxial view.

Due to the size and anatomical location of the cyst (Figure 1), a marsupialization approach was selected as the preferred treatment strategy. A small incision was made for initial biopsy and drainage of the cystic fluid, followed by thorough irrigation with sterile normal saline to cleanse the site. The cystic lining margins were carefully sutured using 3-0 VICRYL*^®^* RAPIDE (polyglactin 910) sutures (Ethicon, Johnson & Johnson Medical Technologies, Cincinnati, OH, USA) to the adjacent gingival mucosa to create a continuous epithelial-lined cavity, preventing premature closure. A rubber drain was placed to ensure continuous decompression and facilitate the gradual evacuation of residual fluid, controlling shrinkage of the lesion. The patient was instructed to perform cyst irrigation via the rubber drain three times daily. A structured follow-up protocol was implemented, comprising weekly visits for the initial four weeks, followed by a six-week assessment. Antibiotic prophylaxis consisted of Amoxicillin 500 mg (Teva Pharmaceuticals Industries Ltd., Petah Tikva, Israel) administered three times daily for one week. To control analgesia, a nonsteroidal anti-inflammatory drug Etodolac^®^ 400 mg (Taro Pharmaceuticals, Haifa, Israel) was prescribed up to three times daily. To minimize infection risk, chlorhexidine 2% (Tarodent*^®^* 0.2% Chlorhexidine Gluconate Oral Rinse, Taro Pharmaceutical Industries Ltd., Haifa, Israel) oral rinses were recommended three times daily for one week. The histopathological analysis, completed three weeks post-procedure, showed a diagnosis of inflamed non-keratinizing odontogenic cyst. At the six-month mark, the patient requested a revision of the treatment plan due to the prolonged duration of therapy and changes in personal circumstances. Therefore, an evaluation was performed, including clinical assessment and radiographic analysis (Figure 2).

The findings indicated favorable cyst regression and improved bone density in the affected area. Based on these results, it was determined that the marsupialization had created optimal conditions for the next phase of treatment. Consequently, the decision was made to proceed with cyst enucleation, marking the transition to the definitive surgical management of the lesion.

### 2.3. Enucleation and Augmentation of the Cystic Defect

**Figure 3:**
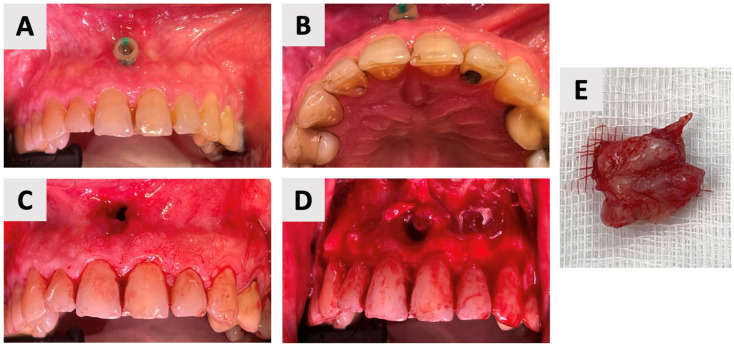
Clinical stages of cyst management and removal. (**A**) Intraoral view showing the rubber drain in situ just before the end of marsupialization. (**B**) Palatal view demonstrating resolution of palatal swelling. (**C**) Surgical flap design with intrasulcular incisions from right to left maxillary canines and vertical releasing incisions at the transitional line angles. (**D**) Intraoperative view after full thickness mucoperiosteal flap elevation, revealing the bony entry point of the rubber drain. (**E**) Clinical view of the completely enucleated cyst specimen.

The surgical phase of cyst enucleation (Figure 3) was initiated following the six-month marsupialization period. Local anesthesia was administered using Cook-Waite*^®^* Lidocaine HCl 2% and Epinephrine 1:100,000 Injection (Septodont, Saint-Maur-des-Fossés, France). The surgical approach began with intrasulcular incisions extending from the right to left maxillary canines. These were complemented by vertical releasing incisions at the transitional line angles of both maxillary canines, facilitating optimal flap design and access (Figure 3C). A full thickness mucoperiosteal flap was elevated, exposing the bony architecture and revealing the entry point of the previously placed rubber drain (Figure 3D).

Complete enucleation of the cystic tissue was performed using specialized sinus augmentation curettes (Kohler Medizintechnik GmbH, Stockach, Germany), ensuring thorough removal of all pathological tissue while preserving surrounding vital structures. Following enucleation, the surgical site was thoroughly irrigated with sterile saline to remove any debris and assess for any remaining cystic tissue. The excised specimen (Figure 3E) was immediately fixed in 10% neutral buffered formalin and sent for histopathological examination to confirm the diagnosis.

**Figure 4:**
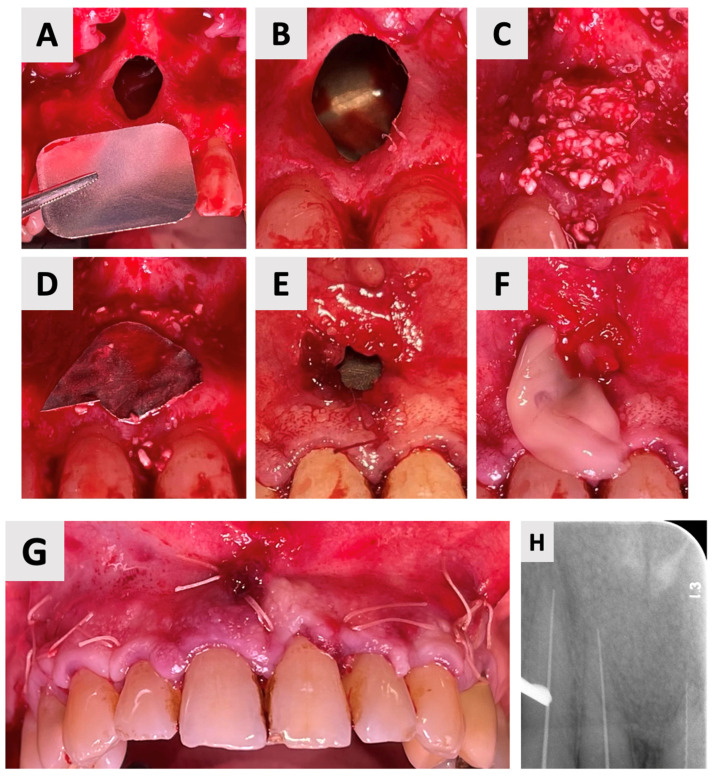
Augmentation of cystic defect: (**A**) Following soft tissue retraction (full mucoperiosteal flap from canine to canine with vertical incisions beginning at the distal transitional line angle of the maxillary canines extending beyond the esthetic zone) to expose the osseous defect, a resorbable magnesium membrane was prepared for placement within the defect cavity. (**B**) The magnesium membrane is carefully positioned at the base of the defect, acting as a structural support to bridge the bony discontinuity in the palatal bone. (**C**) The defect is then filled and compacted with bovine bone graft granules. (**D**) A secondary magnesium membrane is placed over the entrance of the defect, serving as a barrier to secure the graft material and prevent its displacement. (**E**) The soft tissue flap is repositioned and adapted over the defect site to ensure primary closure. (**F**) A platelet-rich fibrin (PRF) membrane is prepared and placed over the soft tissue defect to promote wound healing and soft tissue regeneration. (**G**) Final flap adaptation and primary closure achieved using 3-0 resorbable polyglactin sutures (VICRYL*^®^* RAPIDE, Ethicon, Johnson & Johnson Medical Technologies, Cincinnati, OH, USA). (**H**) Intraoperative retroalveolar radiograph focusing on the apical region of the maxillary incisors, demonstrating satisfactory bone graft condensation within the defect and the working length preparation of the root canals. The post-operative medication regimen followed the same protocol as described after the marsupialization procedure, including antibiotic prophylaxis, analgesic medication, and chlorhexidine oral rinses. A structured follow-up protocol was established, comprising weekly visits for the initial four weeks, followed by a comprehensive assessment at six weeks post-surgery.

Given the size of the resulting osseous defect, it was important to ensure stabilization with a bone graft to prevent the collapse of surrounding vital structures and to promote optimal bone regeneration. An additional challenge was the fact that the cyst had extensively eroded and destroyed part of the palatal bone wall, leaving only a thin layer of palatal soft tissue separating the oral cavity from the underlying bony defect. This anatomical deficiency was a limitation, as it is well known that successful graft compaction and stabilization require a firm structural base. To address this issue, a resorbable magnesium metal membrane (NOVAMag^®^, botiss GmbH, Zossen, Germany) was carefully positioned within the defect to serve as a biomechanical scaffold, effectively reconstructing the firm base of the cavity (Figure 4B). Following the placement of the magnesium membrane to reconstruct the defect base, the cavity was filled with anorganic bovine bone xenograft (cerabone^®^, botiss GmbH, Zossen, Germany). A second magnesium membrane was then carefully positioned over the filled defect, serving as a barrier to prevent soft tissue ingrowth and to GBR (Figure 4E). Then, to improve soft tissue healing at the site where the marsupialization drain had been placed, a platelet-rich fibrin (PRF) membrane was prepared and applied (Figure 4F). Root canal treatment was performed during this phase based on the absence of clinical symptoms and signs of infection during the marsupialization phase. Therefore, following the completion of the bone grafting procedure, immediate root canal treatment was performed using gutta-percha and an epoxy resin-based sealer (AH Plus*^®^*, Dentsply Sirona, Bensheim, Germany) for the root canal obturation. A retroalveolar post-procedure radiograph obtained immediately after the augmentation and endodontic procedures revealed satisfactory bone graft condensation within the defect and adequate endodontic filling of the treated teeth (Figure 4H).

### 2.4. Histopathological Findings

**Figure 5:**
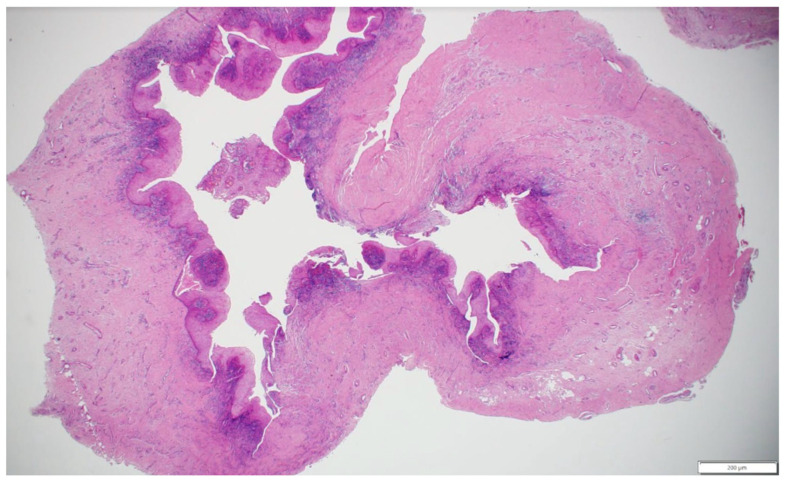
Histopathological features of the excised periapical inflammatory cyst, showing non-keratinized odontogenic epithelium with squamous metaplasia and “hobnail” cells, a thick collagenous wall with chronic inflammatory infiltrate, and prominent vasculature and nerve fibers in the connective tissue (hematoxylin and eosin stain, scale bar = 200 μm).

Histopathological analysis (Figure 5) of the excised cystic lesion revealed a cystic structure lined by hyperplastic, non-keratinized odontogenic epithelium exhibiting squamous metaplasia. Several luminal epithelial cells displayed a characteristic “hobnail” appearance, demonstrating epithelial remodeling. The connective tissue wall was thick and highly collagenous, with chronic inflammatory infiltrates predominantly localized adjacent to the epithelial lining. Additionally, the connective tissue contained multiple large blood vessels and several nerve fibers. These findings, along with the clinical and radiographic correlation, confirm that the lesion represents a periapical inflammatory cyst arising from a non-vital tooth.

### 2.5. Bone and Soft Tissue Healing Follow up

**Figure 6:**
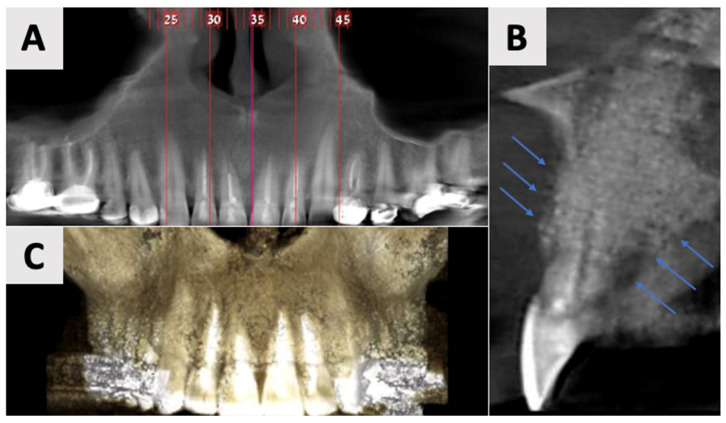
CBCT imaging analysis 2 months post-augmentation. (**A**) Panoramic reconstruction showing the overall healing of the defect site. (**B**) The restored contour of the facial and palatal bony walls (blue filled lines). (**C**) Paraxial view showing the initial osseous regeneration.

The follow-up examinations at 2 and 16 months post-augmentation revealed favorable bone and soft tissue healing outcomes. CBCT scans were performed at both time points. At the 2-month follow-up, CBCT imaging demonstrated initial bone formation within the augmented site (Figure 6).

**Figure 7:**
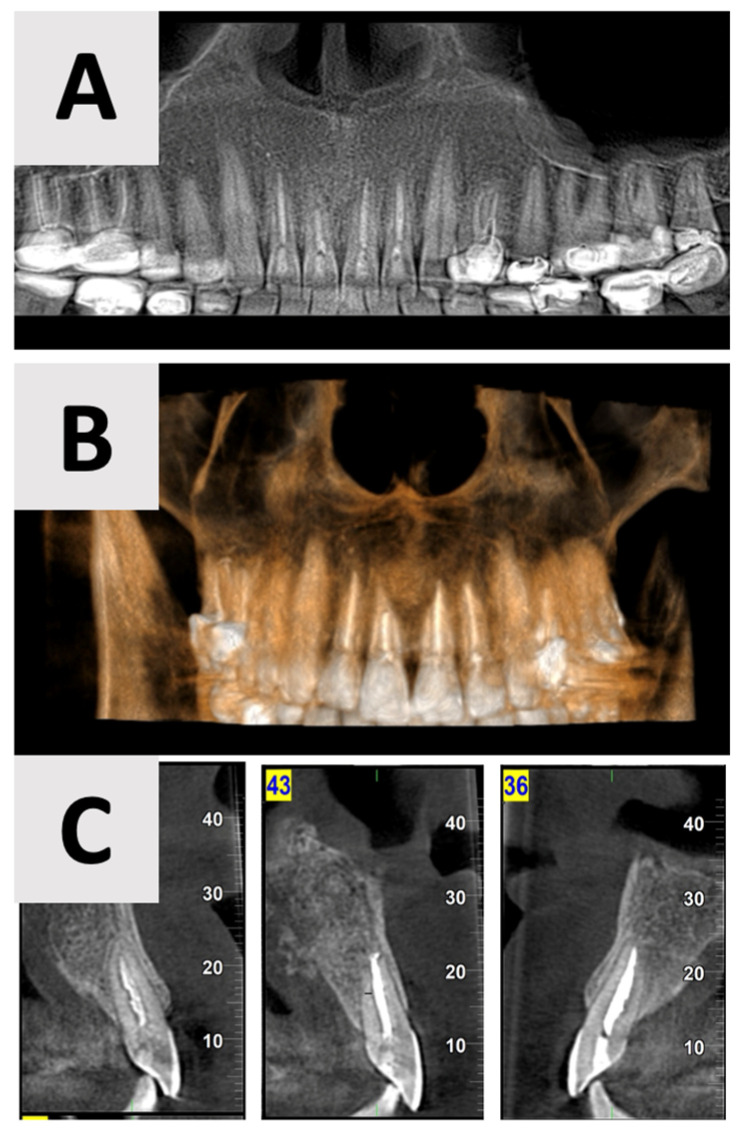
CBCT evaluation of bone regeneration at 16 months post-treatment. (**A**) Panoramic reconstruction showing the absence of radiolucency in the region of prior cyst drainage, indicating a complete osseous integration. (**B**) Three-dimensional paraxial reconstruction illustrating comprehensive bone regeneration throughout the defect site, with restoration of anatomical contours. (**C**) Serial axial CBCT slices (through the affected teeth) demonstrate complete reconstruction of both buccal and palatal bony walls, characterized by the presence of distinct cortical bone layers.

By the 16-month mark, significant improvements in bone volume and density were observed (Figure 7). The palatal contour was restored to its proper anatomical form, with notable cortication of the palatal wall. Importantly, there was no evidence of pathological recurrence around the treated teeth. The buccal entry point for the previously placed rubber dam had healed completely, with cortical bone formation.

**Figure 8:**
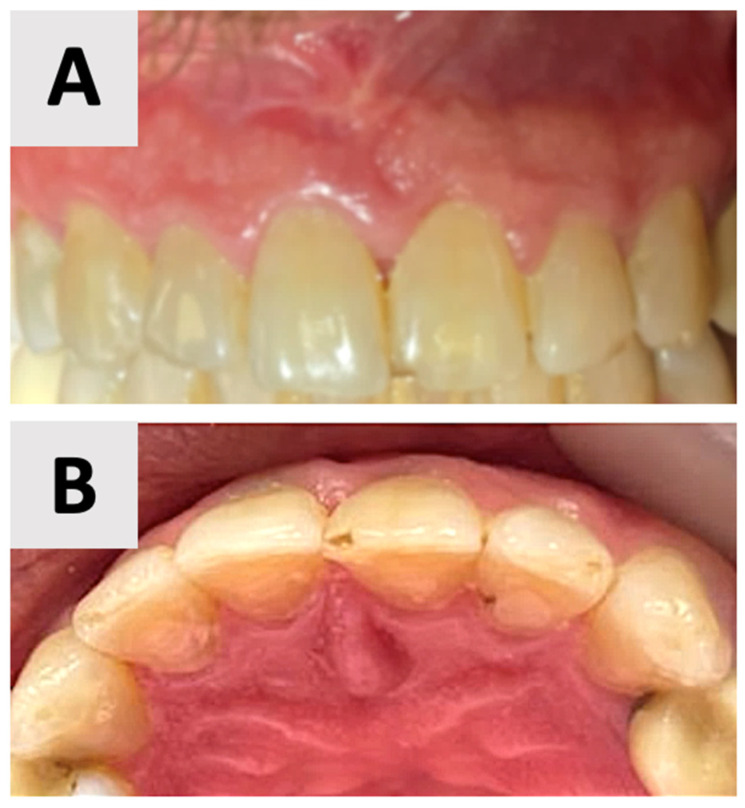
Clinical assessment at 16 months post-augmentation. (**A**) Facial view demonstrating optimal esthetic outcomes with well-preserved gingival architecture and natural tooth appearance. (**B**) Palatal view showing complete soft tissue healing and maintenance of anatomical contour.

Clinical examination at 16 months post-augmentation revealed that the patient remained asymptomatic. The treated teeth exhibited no mobility. Both the alveolar bone and soft tissue contours were well-maintained, preserving the site’s architecture (Figure 8). The patient reported no functional limitations or esthetic concerns, further confirming the success of the procedure from both clinical and patient-centered perspectives.

## 3. Discussion

The treatment of cystic lesions often results in large bone defects, presenting a significant challenge for management following cyst enucleation. Grafting of large bone cavities can provide essential structural support, improve treatment outcomes, and create optimal conditions for osteogenesis [24]. Therefore, in this case report, the combination of a resorbable magnesium membrane and bovine xenograft was evaluated through radiological and clinical assessment as a novel biomaterial approach to improve bone regeneration and stabilize the defect after cyst enucleation.

Bone healing occurs through three general stages that are overlapping: inflammation, bone formation, and finally, bone remodeling. Inflammation starts immediately after the bone is damaged and lasts for several days. The proliferative phase follows. Membranous bone healing occurs with bone production beginning by apposition of a soft matrix around the bony defect site, which is later ossified. Finally, bone remodeling occurs, where the newly formed bone is reshaped, restoring its strength and function [25]. In the context of cystectomy, particularly when one or more bony walls are missing due to a large defect, the healing process may be compromised by soft tissue invasion. Consequently, the surrounding soft tissue infiltrates the defect site, potentially compromising the quality and quantity of newly formed bone [26]. In this case report, we demonstrated how the form-stable magnesium membrane serves as an ideal solution for addressing these requirements. Its structural integrity plays a crucial role in supporting large bone defects such as the defect following cystectomy presented in this case series. The mechanical stability of the membrane helps maintain the shape of the augmented site, providing a stable environment for regeneration. Elad et al. [19] were the first to report the clinical use of the magnesium membrane and introduced the magnesium membrane shield technique as a novel treatment approach for compromised extraction sockets. Their case series demonstrated how the membrane could be applied to rebuild both buccal and palatal walls, allowing immediate implant placement with provisional restoration in the esthetic zone. In their reported cases, satisfactory bone regeneration and soft tissue healing were achieved, with thick cortical bone formation clearly visible in CBCT images, which is indicative of bone regeneration and post-treatment remodeling. Their work helped establish this technique as a viable alternative for ridge preservation and immediate implant protocols. However, their focus was primarily on immediate implant placement, which involves different healing dynamics and structural demands compared to the treatment of the large cystic defect. Also, a recent case by Blašković et al. [20] further supported the application of the magnesium membrane shield technique for alveolar ridge preservation, specifically in a case involving complete buccal wall dehiscence. In their report, a combination of bovine and autologous bone grafts was used, with a magnesium membrane to reconstruct the ridge following tooth extraction. Although their findings included valuable histological evidence of new bone formation, their clinical scenario differed from ours in that it was limited to a single-rooted site post-extraction. In contrast, our case involved a large, multi-rooted radicular cyst with extensive palatal and buccal bone destruction. Additionally, in our case, the natural teeth were preserved rather than extracted. Unlike typical ridge preservation cases, cystic lesions often result in larger and more irregular defects that require extensive structural reconstruction. In our case, the magnesium membrane provided essential mechanical support, prevented soft tissue ingrowth, and contributed to favorable bone healing, as confirmed by CBCT at 16 months.

The gradual resorption of magnesium membranes releases bioactive magnesium ions, which stimulate new bone formation in multiple directions. Namely, magnesium-based biomaterials have great potential in supporting this process, particularly through their ability to support neovascularization and osteoblast activity [27]. Magnesium ions have been shown to stimulate endothelial cell proliferation and migration, promoting angiogenesis, which is essential for delivering oxygen and nutrients to the site of the bone regeneration [28,29]. These positive benefits of vascularization also optimize conditions for osteogenesis. Finally, magnesium exhibits direct osteoinductive properties, influencing osteoblast differentiation, proliferation, and extracellular matrix deposition [30,31]. This makes magnesium membrane a promising material for large post-cystectomy defects, addressing both structural and biological requirements for effective bone healing.

Despite the widespread use of bone grafting techniques, the question of whether to allow spontaneous bone regeneration or use grafting materials to support healing remains controversial in the literature. Currently, some studies have reported comparison of the spontaneous bone healing and bone grafting and concluded that bone density increased over time even without the use of bone grafts [32,33,34]. However, this is not consistent across all cases. For instance, a recent study by Shi et al. found that spontaneous bone formation was not uniformly efficient, particularly in larger defects, where insufficient regeneration and volume shrinkage were observed [35]. This suggests that predictability of bone healing may depend on factors such as defect size, patient-related variables, and the choice of biomaterials.

The present case emphasizes a combined approach to managing a large periapical cystic lesion, involving both surgical and endodontic interventions. The literature often describes non-surgical endodontic therapy as the first line of treatment for periapical lesions [36]. This aligns with recent case reports that advocate for conservative management of large periapical cyst-like lesions [37]. This study reported successful treatment of two cases of large cyst-like periapical lesions in upper central incisors using primarily endodontic measures. Decompression was only employed in one case where canal drying was challenging after three weeks of medication. Our case, however, presented challenges that required a more invasive approach. The extensive size of the lesion, the erosion of the palatal bone wall, and the need for immediate structural support to prevent soft tissue collapse into the defect justified the decision for surgical intervention. Therefore, tailoring the treatment approach to the specific characteristics of each lesion, such as lesion size, bone integrity, proximity to vital structures, and the potential for spontaneous healing is necessary to optimize treatment outcomes, minimize patient discomfort, and create the most favorable conditions for periapical healing. Beyond the conventional approaches discussed, ongoing research is exploring adjunctive regenerative options such as autologous platelet concentrates, stem-cell-based therapies, and next-generation bioresorbable membranes. Although promising, these strategies require further validation through well-controlled clinical studies before they can be widely recommended for routine use [38,39,40].

The use of CBCT has revolutionized the diagnosis, treatment planning, and follow-up of large periapical lesions. In comparison to traditional periapical radiographs, CBCT offers superior characteristics in assessing lesion characteristics and monitoring healing progress [41,42]. CBCT examination allows for more accurate measurements of lesion boundaries in all three planes, providing a comprehensive view of the defect’s extent and its relationship to surrounding anatomical structures. In our case, CBCT imaging revealed the true size of the lesion and the extent of palatal bone wall erosion, factors that were crucial for our treatment plan (Figure 1). The healing process of periapical lesions with bone destruction follows a predictable pattern, starting from the periphery and progressing towards the center [43]. This healing is characterized by a gradual reduction in lesion size due to new bone formation. Complete healing, including the restoration of the periodontal ligament architecture around all root contours, can take anywhere from one to four years [44,45]. In our case, follow-up CBCT examinations at 2 and 16 months (Figure 7 and Figure 8) post-treatment confirmed this staged progress in bone regeneration. The 2-month CBCT showed initial bone formation within the augmented site, while the 16-month scan demonstrated improvements in bone volume and density, with the restored palatal contour and cortication of the palatal wall.

This case report demonstrates the innovative surgical approach using a magnesium membrane in indication of a large periapical cyst; however, it remains an observation with limitations typical of single-case reports. Therefore, the results cannot be generalized, and there is always a risk of over-interpretation without evidence of a clear cause-effect relationship. Further observational and randomized controlled clinical studies are needed to confirm the efficacy, safety, and long-term outcomes of described surgical approach. These studies should compare the efficacy of magnesium membrane in combination with bovine xenograft, with traditional treatment modalities and biomaterials. Also, establishment of clear guidelines for case selection, considering factors such as lesion size, bone integrity, and patient-specific variables, should be the priority for future study designs.

## 4. Conclusions

Our findings indicate that a combined approach involving marsupialization, enucleation, and GBR with a magnesium membrane and bovine xenograft may be a promising strategy for managing large periapical cystic lesions, though further studies are needed to confirm its broader applicability. While our treatment was more invasive than conservative endodontic therapy alone, it demonstrated several benefits because of extensive bone loss. Therefore, this approach may be of interest, especially in complex cases where non-surgical methods are insufficient or impractical. However, as per the literature, it is important to note that a non-surgical approach should always be considered as the first line of treatment for endodontic origin cystic lesions, when possible, due to their minimally invasive nature and high success rates. The decision to choose more extensive surgical interventions should be based on careful consideration of individual case characteristics, like in this described case report, including lesion size, bone integrity, and the potential for spontaneous healing.

## Data Availability

The original contributions presented in the study are included in the article.

## Supplementary Materials

The following supporting information can be downloaded at: https://www.mdpi.com/article/10.3390/healthcare13091068/s1, CARE Checklist.
