# Supplementary material for: Use of a Resorbable Magnesium Membrane for Bone Regeneration After Large Radicular Cyst Removal: A Clinical Case Report
Source: Healthcare (Basel). 2025 May 6;13(9):1068. doi: 10.3390/healthcare13091068 (PMC12071955; doi:10.3390/healthcare13091068)
Supplement: Supplementary file 1 [file healthcare-13-01068-s001.zip › healthcare-3582049-supplementary.pdf]

CARE Checklist of information to include when writing a case report

CARE case report guidelines

(cc) BY-NC-ND

Topic	Item	Checklist item description	Reported on Line
Title	1	The diagnosis or intervention of primary focus followed by the words "case report"	1-4
Key Words	2	2 to 5 key words that identify diagnoses or interventions in this case report, including "case report"	42-43
Abstract (no references)	3a	Introduction: What is unique about this case and what does it add to the scientific literature?	23-27
	3b	Main symptoms and/or important clinical findings	27-28
	3c	The main diagnoses, therapeutic interventions, and outcomes	28 - 35
	3d	Conclusion—What is the main "take-away" lesson(s) from this case?	35 - 41
Introduction	4	One or two paragraphs summarizing why this case is unique (**may include references**)	103-112
Patient Information	5a	De-identified patient specific information	121
	5b	Primary concerns and symptoms of the patient	121-128
	5c	Medical, family, and psycho-social history including relevant genetic information	122 - 124
	5d	Relevant past interventions with outcomes	125 - 128
Clinical Findings	6	Describe significant physical examination (PE) and important clinical findings	129 - 141
Timeline	7	Historical and current information from this episode of care organized as a timeline	121 - 150
Diagnostic Assessment	8a	Diagnostic testing (such as PE, laboratory testing, imaging, surveys).	142 - 153
	8b	Diagnostic challenges (such as access to testing, financial, or cultural)	non-applicable
	8c	Diagnosis (including other diagnoses considered)	154-155
	8d	Prognosis (such as staging in oncology) where applicable	non-applicable
Therapeutic Intervention	9a	Types of therapeutic intervention (such as pharmacologic, surgical, preventive, self-care)	155 - 158
	9b	Administration of therapeutic intervention (such as dosage, strength, duration)	159 - 243
	9c	Changes in therapeutic intervention (with rationale)	non-applicable
Follow-up and Outcomes	10a	Clinician and patient-assessed outcomes (if available)	258 - 288
	10b	Important follow-up diagnostic and other test results	258-288
	10c	Intervention adherence and tolerability (How was this assessed?)	259-262; 268
	10d	Adverse and unanticipated events	non-applicable
Discussion	11a	A scientific discussion of the strengths AND limitations associated with this case report	365 - 375
	11b	Discussion of the relevant medical literature **with references**	290 - 375
	11c	The scientific rationale for any conclusions (including assessment of possible causes)	290 - 264

	**11d**	The primary “take-away” lessons of this case report (without references) in a one paragraph conclusion.................	277 - 388
**Patient Perspective**	**12**	The patient should share their perspective in one to two paragraphs on the treatment(s) they received	282 - 284
**Informed Consent**	**13**	Did the patient give informed consent? Please provide if requested	**Yes** ☒ **No** ☐
